# A New Reactive Force Field for NO_*x*_‑Water Interactions: Development, Evaluation, and Molecular Dynamics Simulation of Peroxynitrite

**DOI:** 10.1021/acs.jpcb.6c03657

**Published:** 2026-09-25

**Authors:** Masoom Shaban, Adri C. T. van Duin, Diana van Duin, Margaret Kowalik, Mohammad E. Izadi, Alfred P. Weber, Nina Merkert

**Affiliations:** † Institute of Metallurgy, Clausthal University of Technology, Clausthal-Zellerfeld 38678, Germany; ‡ Institute of Particle Technology, 26534 Clausthal University of Technology, Clausthal-Zellerfeld 38678, Germany; § RxFF Consulting LLC, 1524 West College Avenue, Suite 202, State College, Pennsylvania 16801, United States; ∥ Department of Chemistry, 48437 University of Isfahan, Isfahan 81746-873441, I. R. Iran

## Abstract

Reactive nitrogen species (RNSs) are key contributors to plasma-liquid processes and play a critical role in the chemistry of plasma-activated water (PAW). Despite their importance, their molecular-level behavior in aqueous environments is still not well understood, largely due to the absence of reliable reactive force fields. In this study, a new ReaxFF parameter set, ReaxFF_CHONx_-2026, is developed and systematically assessed for modeling nitrogen- and oxygen-containing reactive species under both gas-phase and aqueous conditions relevant to plasma systems. The parameter set is optimized using density functional theory (DFT) reference data for representative NO_*x*_ reaction pathways and integrated with an existing aqueous/biomolecular ReaxFF framework to enable reactive molecular dynamics simulations in water. Its performance is validated through a range of structural, thermodynamic, and dynamic properties including radial distribution functions, potential energy profiles, and diffusion coefficients. The results demonstrate strong agreement with DFT calculations and previously reported data, particularly in describing N–O bond breaking and solvation behavior. As a case study, the interaction of peroxynitrite (ONOO^–^) with water is investigated using reactive molecular dynamics, where atomic charges are dynamically determined via the ReaxFF charge equilibration method. The simulations show protonation leading to ONOOH formation, pronounced site-specific solvation around oxygen atoms, and preferential cleavage of the peroxide O–O bond. Analysis of diffusion further indicates that the predicted transport properties of RNS are consistent with the available data, supporting the reliability of the developed force field. Overall, ReaxFF_CHONx_-2026 offers a robust framework for large-scale atomistic simulations of PAW and reactive nitrogen chemistry.

## Graphical abstract

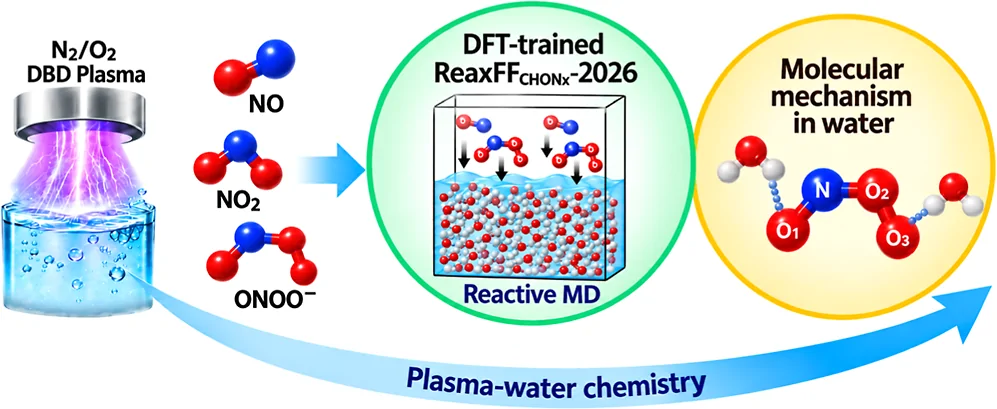

## Introduction

The interaction between reactive species generated in plasma and liquid water is a key subject in plasma science, as it governs both the chemical composition of plasma-activated water (PAW) and the resulting biological responses of plasma-based treatments.^1^ Cold atmospheric plasma produces a broad set of reactive oxygen and nitrogen species (RONS), ranging from short-lived radicals to more persistent molecules such as OH, O_3_, H_2_O_2_, nitric oxide (NO), NO_2_, and ONOO^–^; these species are involved in both gas-phase chemistry and plasma-liquid interfacial reactions.^2,3^ When these species come into contact with water, they dissolve, diffuse, and undergo chemical transformations, generating PAW enriched with reactive species that exhibit strong oxidative and nitrosative properties.^4,5^ These properties have enabled numerous applications in water treatment, food safety, agriculture, and biomedical technologies.^6–8^ In biological contexts, plasma–water interactions are especially important because cells, bacteria, and viruses are typically covered by thin aqueous films.^9^ Consequently, plasma-generated species must diffuse through water before reaching biological targets. The solvation and transformation of reactive species govern their lifetime and reactivity.^10–12^ Therefore, understanding plasma–water interactions at the molecular level is essential for elucidating plasma-liquid chemistry.

At the molecular level, theoretical methods and molecular dynamics (MD) simulations provide effective tools for exploring the processes that control how reactive species interact with water. Among these approaches, reactive molecular dynamics simulations based on the ReaxFF force field are particularly powerful, as they account for bond formation and breaking, enabling the investigation of radical-driven chemistry in aqueous environments.^13^ Building on this capability, in our previous work, ReaxFF-based MD simulations were used to examine the interaction of reactive oxygen species (ROS) (OH and H_2_O_2_) with water, providing atomistic insight into plasma-liquid interfacial processes.^14^ In addition to this work, several studies have employed ReaxFF to investigate the interaction of ROS with water. For example, reactive MD simulations examined the interaction of oxygen radicals with a liquid water layer and demonstrated how species such as O° and OH° react with water molecules and initiate oxidative reaction cascades within the liquid phase.^9^ In addition, quantum-based MD simulations have shown that atomic oxygen can react with water to form hydrogen peroxide via multiple pathways, including hydrogen abstraction and proton-transfer mechanisms.^15^ Further ReaxFF-based simulations have revealed hydrogen-abstraction mechanisms of hydroxyl radicals and the formation of secondary reactive species in aqueous environments.^16^ Similarly, atomistic simulations have highlighted the important role of hydrogen-bond networks in modulating radical transport and reactivity in water.^17^

Besides reactive force-field methods, the behavior of reactive species in water has also been examined with classical nonreactive force fields and quantum-chemical calculations.^18^ Molecular dynamics simulations using a combined quantum/classical force field have been applied to investigate the solvation structure and diffusion behavior of hydrogen peroxide (H_2_O_2_) in aqueous environments, capturing both electronic effects and intermolecular interactions.^19^ For instance, in a recent study, a classical, nonreactive all-atom force field was developed for NO and its dimer ((NO)_2_), based on Lennard-Jones and Coulombic interactions, to reproduce vapor–liquid equilibrium properties and dimerization behavior.^20^ These studies showed that NO interacts only weakly with water, causing little disturbance to the hydrogen-bond network, while quantum-chemical calculations likewise identified mainly weak van der Waals and electrostatic interactions in the H_2_O–NO complex.^21,22^ Recent cross-scale QM/MM studies have provided detailed descriptions of localized reactions involving reactive species in aqueous and interfacial environments. For example, Xu et al.^23^ employed adaptive QM/MM molecular dynamics to investigate plasma-generated atomic oxygen at a gas–liquid interface, while recent QM/MM simulations have also been used to examine peroxynitrite reactions in aqueous solution.^24^ Although QM/MM substantially extends the accessible system size compared with fully quantum simulations, its computational cost and the need to define or dynamically update the QM region generally restrict continuous reactive sampling over extended time scales. In contrast, reactive force-field simulations enable system-wide bond rearrangement and nanosecond-scale dynamic evolution without predefining a localized reactive region.

Furthermore, ab initio MD simulations based on density functional theory (DFT) have been applied to investigate reactive nitrogen species (RNSs) in different environments. For example, studies on the OH° + NO_2_° system demonstrated a barrierless reaction pathway leading to the formation of HONO_2_ and ONOOH.^25^ Similarly, first-principles MD simulations of peroxynitrous acid in aqueous solution revealed that only cis and trans conformers are stable, with their relative stability strongly influenced by solvent effects.^26^ In addition, ab initio MD studies of N_2_O_5_ hydrolysis on water clusters showed rapid formation of nitric acid through transition-state-driven reaction pathways.^27^

Although these methods have provided useful atomistic information, earlier work has mainly addressed oxygen-centered reactive species, whereas nitrogen-containing species have usually been studied with nonreactive force fields or ab initio approaches. However, nonreactive models cannot capture bond-breaking processes, and ab initio simulations are limited to small systems and short time scales, restricting their applicability to complex plasma-liquid environments. In addition, the chemical complexity of NO, NO_2_, peroxynitrite (ONOO^–^), and their aqueous derivatives further complicates accurate modeling, particularly in the absence of reliable reactive force fields for these systems. In this work, we address this gap by developing a new reactive force field (ReaxFF_CHONx_-2026) for nitrogen-containing species and applying it to investigate the interaction of key plasma-generated species, peroxynitrite, with water at the molecular level. This framework provides a foundation for ongoing extensions to other NO_*x*_ systems, including NO_2_, N_2_O_5_, and their coupled reaction networks, enabling large-scale simulations of PAW chemistry.

## Methods

### Parameterization of the ReaxFF_CHONx_-2026

To describe the reactive behavior of nitrogen- and oxygen-containing species in water, we used the ReaxFF reactive force field,^28^ which can represent bond formation and bond cleavage in chemically complex systems. This capability is essential for simulating the intricate chemistry of RONS that are relevant to plasma-liquid interactions.

ReaxFF has been applied successfully in many reactive systems, but the available parameter sets were developed mainly for hydrocarbon-, biomolecular-, and oxygen-centered chemistries.^29^ For instance, the ReaxFF versions developed by Monti et al.^30^ and Rahaman et al.^31^ focused on amino acids, peptides, and phosphorus oxides, with reactive species such as OH, H_2_O_2_, and HO_2_ playing central roles.^16,17,30,32^ Nevertheless, nitrogen (NO, NO_2_, ONOO^–^) and its aqueous reaction products (HNO_2_, HNO_3_) were not incorporated into the training sets of these force fields, limiting their ability to accurately capture NO_*x*_ interactions with water. In particular, bond-breaking and -forming processes between these species and water molecules are not accurately described. To overcome these limitations, we developed a new ReaxFF parameter (ReaxFF_CHONx_-2026) to simulate NO_*x*_–water interactions, enabling chemically accurate and mechanistically realistic simulations of their reactivity in aqueous environments.

To improve the ReaxFF accuracy for plasma composition modeling, especially modeling the chemistry characteristic for the RNSs, a series of the reactions involving the NO_*x*_ radicals, characteristic for the gas as well as the aqueous phase, were considered in optimizing the ReaxFF parameter set. Starting with the ReaxFF parameters published by Wood et al.,^33^ the gas-phase reactions that produce NO and NO_2_ radicals, in particular reactions of O radicals with N_2_, N radicals with O_2_, as well as reactions with OH radicals and ozone with NO, were considered. The ReaxFF reaction energy barriers were determined and compared to the same reaction barriers obtained with the use of the DFT calculations. Subsequently, the ReaxFF_CHON*x*_-2026 parameter set was optimized, if necessary, to minimize the difference between the DFT and ReaxFF data. All the DFT data were obtained with the use of ADF as implemented within the Amsterdam modeling suite (AMS) software and the GGA/PBE-D3 functionals along with the TZP basis set.^34,35^

The newly optimized ReaxFF_CHONx_-2026 parameters for the gas phase were further merged with the ReaxFF description for peptides and lipid bilayers previously published by Monti et al.^30^ and further optimized for the NO_*x*_ radical, anion, and neutral reactions in the aqueous phase, which include HNO_2_, HNO_3_, NO_2_^–^, NO_3_^–^, their complexes with water as well as their reactions with water, with peptides, and model lipid structures. All considered reaction energies are listed in Table S1, while the comparison of the reaction energies is shown in Figure 1. Several reaction pathways, such as NO_2_ + 2H_2_O conversion to HNO_3_ and H_3_O, see Figure S1, were also taken into account in the ReaxFF parameter set optimization process.

**Figure 1:**
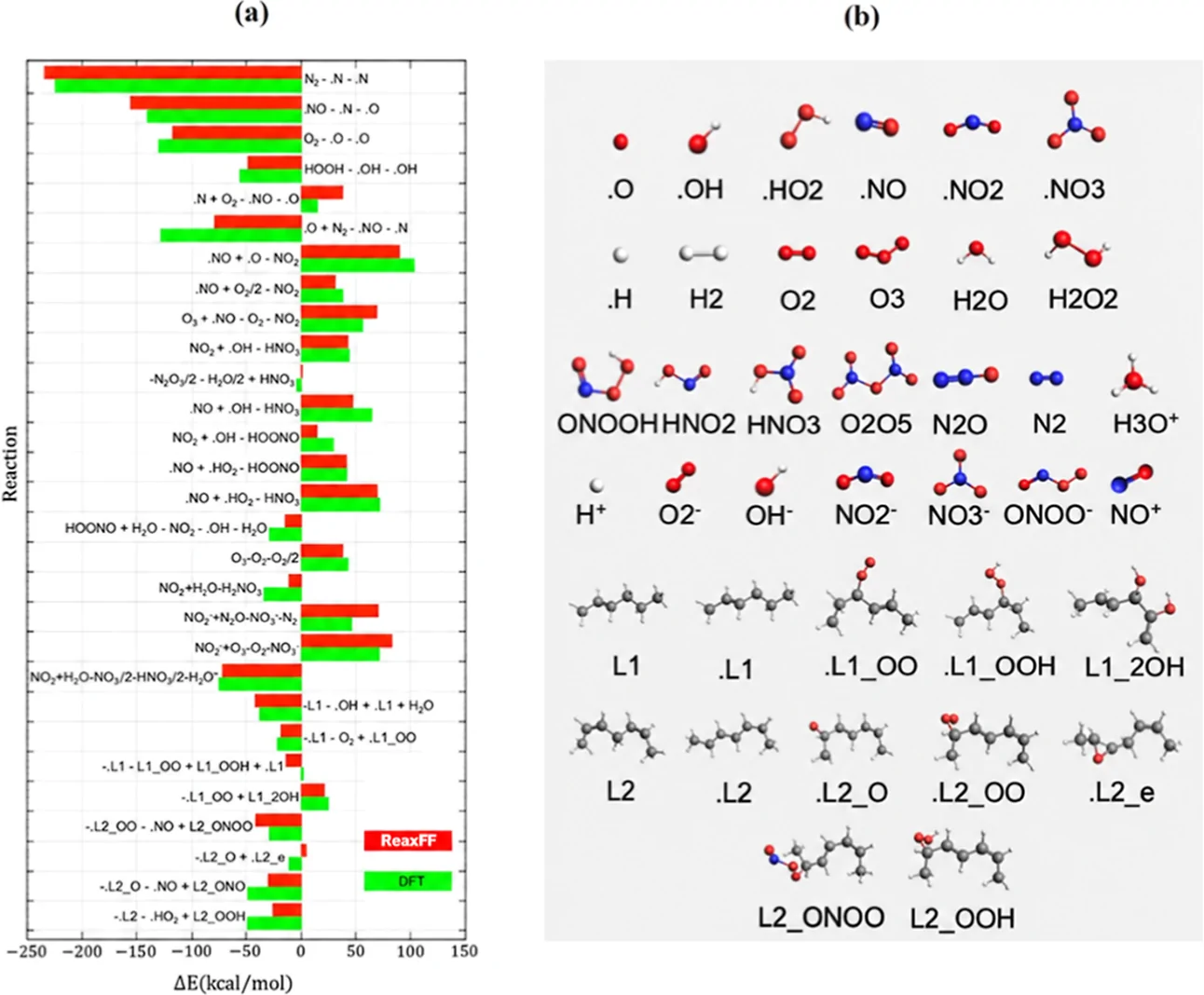
(a) A comparison of the reaction energies obtained based on the DFT versus ReaxFF_CHONx_-2026 calculations. (b) The snapshots of all considered structures, where L1/L2 represents a model alkene chain with one or two unsaturated C–C bonds, respectively.

### Setup of Molecular Dynamics Simulations

All molecular dynamics calculations were performed with LAMMPS,^36^ which propagates atomic trajectories by numerically integrating Newton’s equations of motion. OVITO Pro was used for visualization and postprocessing to analyze trajectories, bond evolution, and the spatial distribution of reaction products. Building upon the newly developed ReaxFF_CHONx_-2026 potential, a representative simulation setup was designed to investigate the atomistic mechanisms governing NO_*x*_-water interactions. The simulation model was constructed within a 25 × 25 × 65 Å^3^ box. Periodic boundary conditions were applied along the *X* and *Y* directions to represent an infinite lateral system, while the *Z* direction was fixed to confine the water slab vertically. To prevent translational drift of the liquid slab, the center of mass of the water phase was maintained at its initial position along the nonperiodic *Z* direction throughout the simulation. The lower region (0–25 Å along the *Z*-axis) contained 522 water molecules with an approximate density of 1 g cm^–3^, and the upper region (35–65 Å) included 5–10 NO or ONOO^–^ molecules, depending on the simulation case, separated from the water surface by a 10 Å vacuum gap. This initial spacing ensured that no interactions occurred between the water layer and the NO_*x*_ molecules during the equilibration stage (Figure 2a, snapshot at *t* = 0 ns).

**Figure 2:**
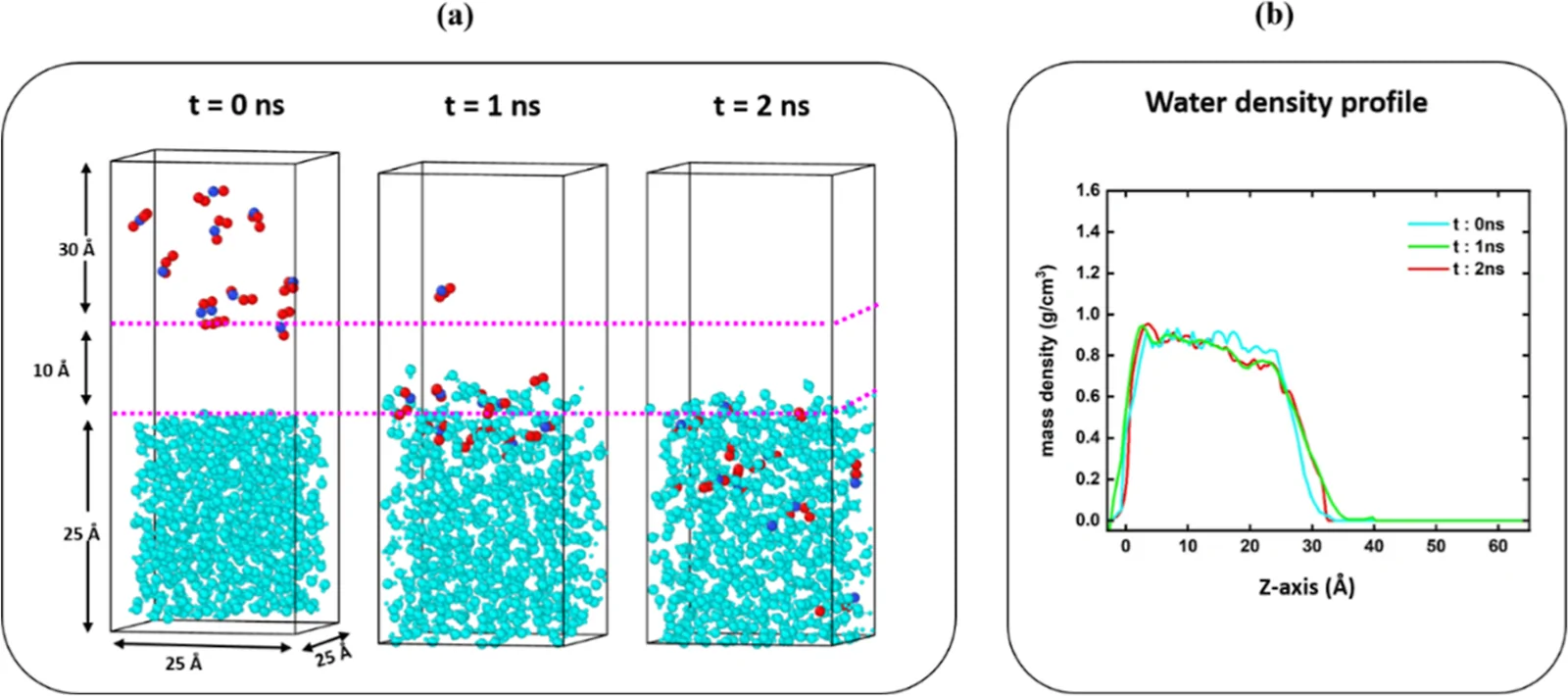
(a) Snapshots of the water-NO_*x*_ system at *t* = 0, 1, and 2 ns, showing ONOO^–^ distribution in the gas phase, interfacial region, and water phase. Nitrogen atoms are shown in blue, oxygen atoms in red, and water molecules in cyan for clarity. (b) Water density profiles along the *Z*-axis at the corresponding times, illustrating the liquid–vacuum interface.

A cutoff radius of 10 Å was employed for the nonbonded interactions and was kept constant throughout the simulations. This value is consistent with interaction ranges commonly used in related reactive-force-field simulations. Rahnamoun et al. reported that shielded Coulomb interactions in ReaxFF are smoothly tapered to zero, with typical electrostatic cutoff values of approximately 10–12 Å,^37^ while Yoo et al.^38^ employed ReaxFF-based long-range van der Waals and Coulomb interactions up to 10 Å for reactive CHNO systems. A comparable real-space electrostatic cutoff of 9 Å has also been used for the charged nitrogen oxyanions NO_3_^–^ and NO_2_^–^ at the water–air interface.^39^

To prevent molecular overlap, all species were inserted individually at random positions. Each system was first energy-minimized to remove atomic overlaps and obtain a relaxed configuration, followed by thermalization at 300 K in the canonical ensemble (*NVT*) to achieve structural and thermal equilibrium. Subsequently, the system was equilibrated for 500 ps (0.5 ns) using a Nosé–Hoover thermostat with a coupling constant of 25.0 fs to maintain constant temperature, while the simulation volume remained fixed by definition of the *NVT* ensemble. The equilibrated system was then used for production simulations over a duration of 4 ns to examine the interaction behavior of NO and peroxynitrite species in water. Although peroxynitrite is formally an anion (ONOO^–^), its charge is not imposed as a fixed value in the present simulations. Within the ReaxFF framework, atomic charges are dynamically determined at each time step using a charge equilibration scheme, as described in the original ReaxFF formulation by van Duin et al.,^28^ allowing the negative charge to be distributed over the molecule as time-dependent partial charges.^28,40^ To assess charge stability, the QEq-derived partial charges were monitored throughout the trajectories. For the atoms initially belonging to ONOO^–^, the partial charges remained within a limited range of approximately −0.45 to −0.10 e without systematic drift. The net charge of each original ONOO^–^ group also remained close to its formal −1 charge after equilibration, while the negative charge was distributed over the N–O framework. No abrupt charge changes were observed prior to the reactive event, indicating that the observed reaction was not driven by excessive *Q*_eq_ charge fluctuations. The application of periodic boundaries in the lateral directions and nonperiodic fixed boundaries along the *Z*-axis enabled the formation of a stable liquid–vacuum interface, allowing a realistic representation of interfacial molecular processes.

Figure 2a illustrates the time evolution of the simulation box during the interaction of the NO_*x*_ species with the water slab. At *t* = 0 ns, the water molecules form a compact liquid layer in the lower part of the box, while the NO_*x*_ molecules are initially located above the water surface and are separated by a vacuum region. As the simulation proceeds, the NO_*x*_ molecules gradually approach the interface, and by *t* = 1 and 2 ns several molecules have penetrated into the interfacial and liquid regions, indicating progressive interaction with water. Figure 2b shows the corresponding water mass density profiles along the Z-axis at different simulation times. The density in the bulk liquid region remains close to 1 g cm^–3^ throughout the simulation, confirming that the water slab preserves a physically realistic liquid structure. At the same time, a slight broadening of the interfacial region is observed at longer times, consistent with surface restructuring and the increasing interaction of NO_*x*_ molecules with water.

## Results and Discussion

### Validation of the ReaxFF_CHON*x*_-2026 in Aqueous Systems

The reliability of ReaxFF_CHON*x*_-2026 and the associated water description was evaluated by investigating the representative structural, static, and dynamic properties of the simulated system. As illustrated in Figure 3a–c, the radial distribution functions (RDFs) for O–O, H–H, and O–H pairs obtained using ReaxFF_CHON*x*_-2026 are in excellent agreement with those reported in a previous study by Rahaman et al.^31^ This consistency supports the accuracy of the molecular interactions and structural organization captured in our models.

**Figure 3:**
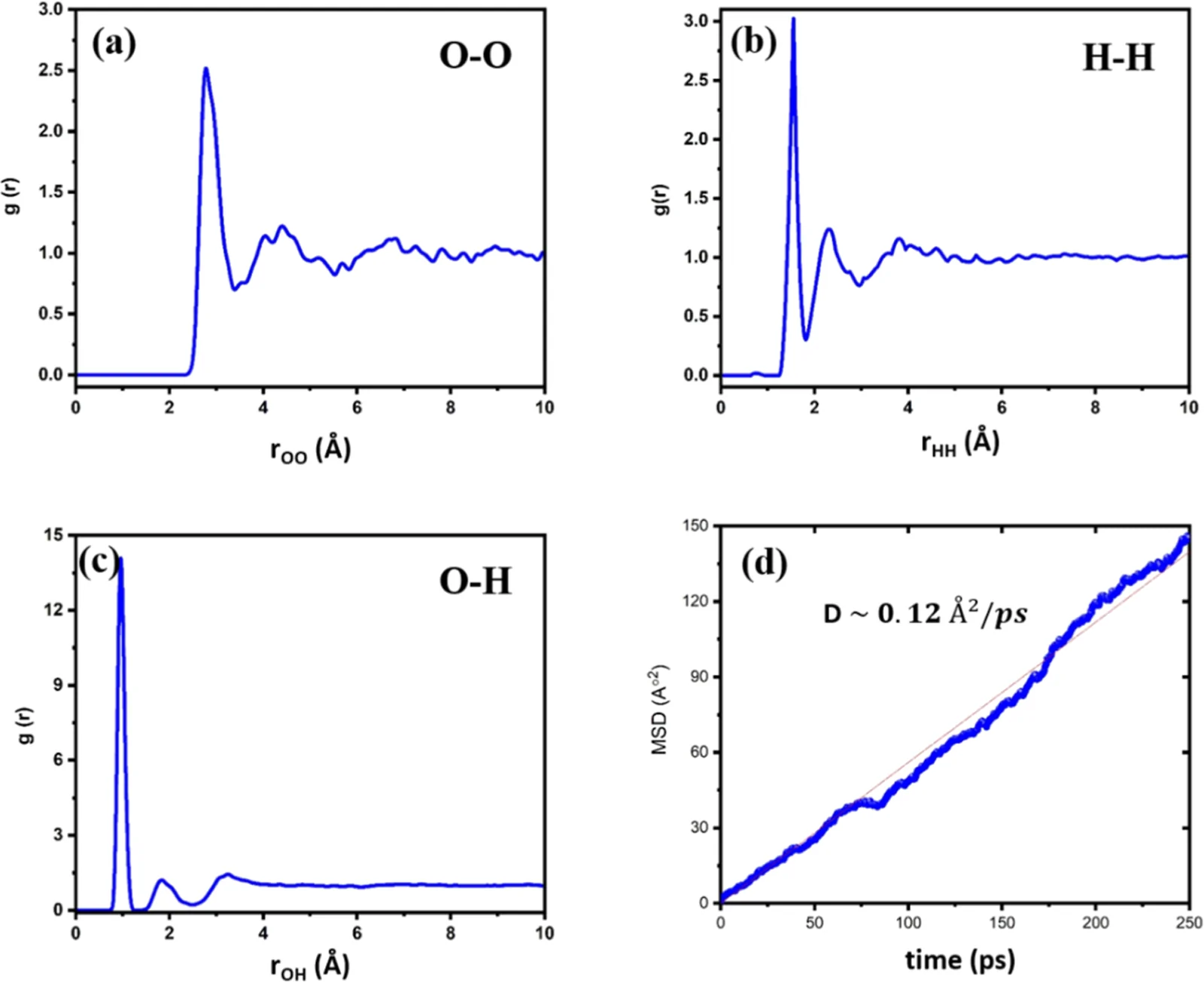
RDFs obtained from MD simulations using the ReaxFF_CHON*x*_-2026 force field: (a) O–O, (b) H–H, and (c) O–H atomic pair correlations in water. (d) Mean squared displacement (MSD) of water molecules, with an estimated diffusion coefficient of 0.12 Å^2^. ps^–1^.

In addition to structural characterization based on RDF analysis, the dynamic behavior of the system was evaluated through the self-diffusion coefficient of water derived from the long-time regime of the MSD, calculated at 300 K using the ReaxFF_CHONx_-2026 force field (Figure 3d), and via the Einstein relation (eq 1).^41^

1 $$D=\frac{1}{6N}\sum_{i=1}^{N}\lim_{t\to \infty }\left\langle \frac{\left\langle {\left|r_{i}\left(t\right)-r_{i}\left(0\right)\right|}^{2}\right\rangle }{t}\right\rangle$$

In this equation, *r*_*i*_(*t*) is the position of atom *i* at time *t* and *N* is the total number of atoms over which the MSD is averaged. The factor of 6 accounts for diffusion in three dimensions.

The calculated diffusion coefficient (≈0.12 Å^2^. ps^–1^) agrees well with previously reported values from ReaxFF simulations by Yusupov et al.^9^ and from ab initio simulation.^42^ It should be noted that the experimentally measured diffusion coefficient of bulk water at room temperature is approximately 0.20 Å^2^. ps^–1^ (2.3 × 10^–9^ m^2^. s^–1^).^43^ The slightly lower diffusion values obtained in molecular simulations are typical and largely attributed to finite-size effects and the intrinsic limitations of empirical potentials.

The accuracy of the developed force field in describing reactive species was further evaluated using the potential energy curve (PEC) of the NO bond and its dynamic behavior in aqueous environments. The NO dissociation profile (Figure 4a) was obtained by comparing ReaxFF predictions with DFT calculations (PBE-D3/TZP) performed using ADF within the AMS.^34^ The DFT reference yields an equilibrium bond distance of *r*_eq_ ≈ 1.16 Å and a minimum energy of approximately −149.6 kcal·mol^–1^, which are consistent with literature values (1.14–1.16 Å and 145–155 kcal mol^–1^).^38^ The ReaxFF_CHON*x*_-2026 model shows close agreement with the DFT results, predicting *r*_eq_ ≈ 1.16 Å and *E*_min_ ≈ −147.9 kcal mol^–1^. In contrast, the earlier parametrizations exhibit noticeable deviations: ReaxFF (Rahaman et al., 2011)^31^ overestimates the bond strength (*r*_eq_ ≈ 1.09 Å and *E*_min_ ≈ −156.1 kcal mol^–1^), while ReaxFF (Monti et al., 2013)^30^ underestimates the well depth (*r*_eq_ ≈ 1.23 Å and *E*_min_ ≈ −141 kcal mol^–1^). Moreover, both previous force fields show an artificial shoulder in the intermediate region (∼1.5–2.5 Å), indicating an inaccurate description of partially stretched bond configurations. This artifact is absent in the ReaxFF_CHON*x*_-2026 model, which yields a smooth and physically consistent dissociation curve in close agreement with DFT. This limitation explains why these earlier parametrizations have primarily been applied to species such as OH, H_2_O_2_, and H_2_O, and are not well suited for accurately describing NO bond dissociation.^9^

**Figure 4:**
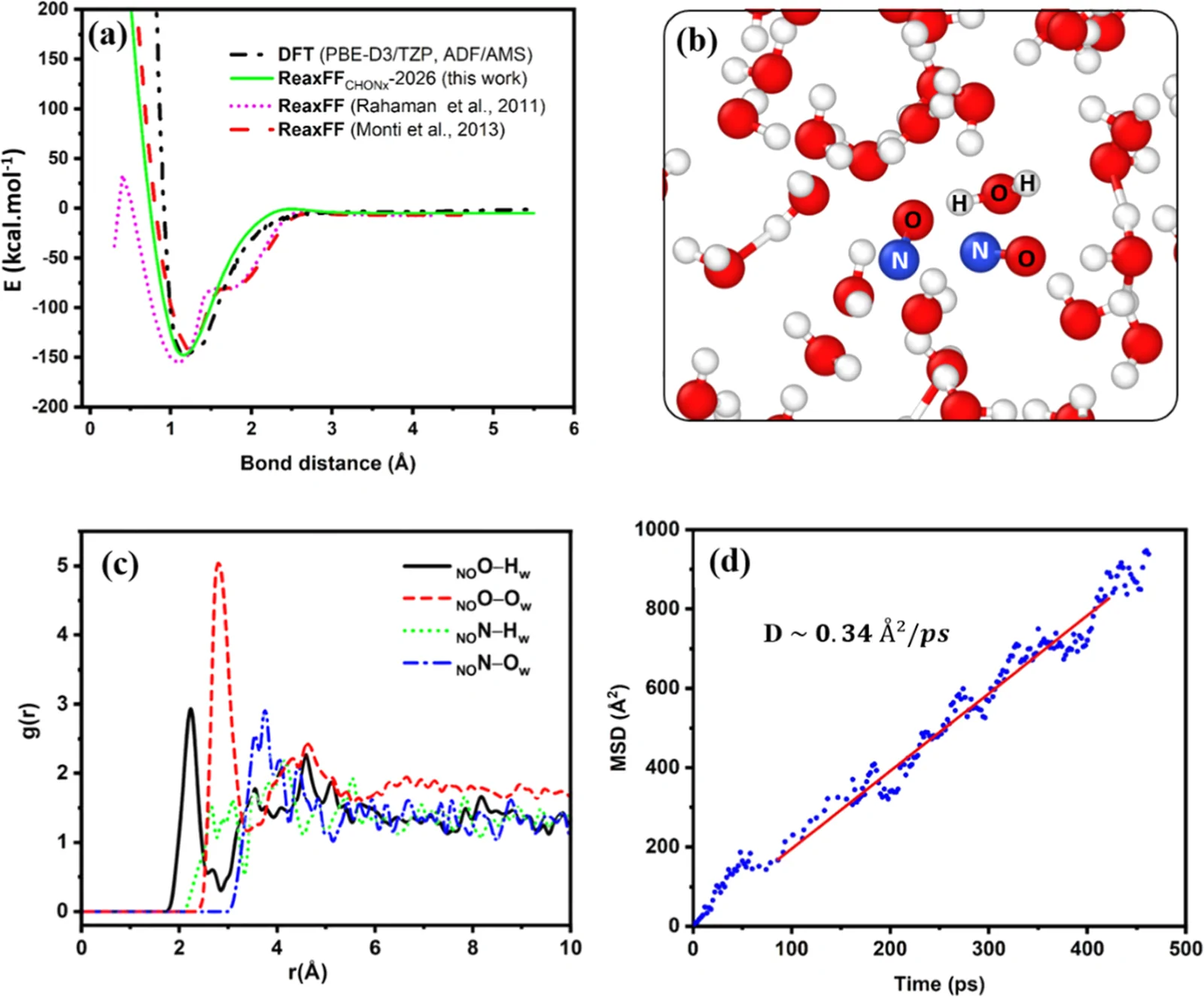
(a) PEC of the N–O bond calculated using ReaxFF (Rahaman et al., 2011 ^31^), ReaxFF (Monti et al., 2013),^30^ ReaxFF_CHON*x*_-2026, compared with DFT results, (b) simulation snapshots of NO molecules in water, (c) RDF of NO-water interactions obtained from MD simulations using the ReaxFF_CHON*x*_-2026, (d) MSD of NO in water, with an estimated diffusion coefficient of 0.34 Å^2^.ps^–1^.

To further evaluate the behavior of NO in aqueous conditions, molecular dynamics simulations were conducted using the previously described setup. As shown in Figure 4b, NO molecules are solvated within the liquid phase. Moreover, RDF analysis of NO in water shows a pronounced intramolecular N–O peak at ∼1.1 Å (Figure S2), confirming the stability of the NO bond throughout the simulation. The intermolecular RDFs (Figure 4c) indicate that solvation is dominated by the oxygen atom of NO, as evidenced by a strong _(NO)_O–O_(water)_ peak located at ∼3.0 Å, while a distinct _(NO)_O–H_(water)_ peak at ∼2.3 Å indicates the presence of close hydrogen-bonding interactions. In contrast, RDFs involving the nitrogen atom are broader and less intense, as shown in Figure 4c by the _(NO)_N–H_(water)_ and _(NO)_N–O_(water)_ peaks being shifted to larger distances (∼3.6–3.8 Å), indicating weaker interactions. Overall, these results show preferential hydration at the oxygen site and confirm that NO remains chemically stable and does not react with pure water under these conditions. These findings confirm that NO is surrounded by a stable and persistent hydration shell governed by weak intermolecular interactions and are consistent with previous theoretical and experimental studies reporting that NO forms only weak hydration complexes in water in the absence of oxidizing species or radicals.^21^

The dynamic properties were further examined through MSD analysis (Figure 4d). A detailed description of how the diffusion coefficient was determined from the molecular dynamics trajectories is presented later in this paper. The diffusion coefficient of NO obtained using the present ReaxFF_CHONx_-2026 force field is approximately 0.34 Å^2^ ps^–1^, which is in good agreement with values reported from classical nonreactive molecular dynamics simulations (∼0.31–0.36 Å^2^ ps^–1^ at ambient conditions).^21^

This agreement indicates that the present force field not only accurately captures the structural and solvation characteristics of NO in water but also reliably reproduces its dynamic behavior. Overall, the combined structural (RDF) and dynamical (MSD) results demonstrate that the ReaxFF_CHON*x*_-2026 parametrization provides a consistent and physically realistic description of NO in aqueous environments while the force field shows clear improvements over previous parametrizations in describing NO bond dissociation and solvation behavior.

### Interaction of Peroxynitrite and Water

The interaction between ONOO^–^ and water was investigated using molecular dynamics simulations, in which ONOO^–^ molecules were initially positioned approximately 1 nm above a water slab, following the simulation setup described previously. To monitor the structural evolution and interaction mechanisms in the aqueous phase, RDFs between ONOO^–^ and water atoms were analyzed. The RDFs at 300 K reveal distinct site-specific solvation and reactivity patterns (Figure 5). The _(ONOO)_O–H_(water)_ RDF exhibits a sharp peak at ∼1.0 Å, indicating the formation of O–H covalent bonds and suggesting proton transfer from water leading to ONOOH formation, consistent with the known protonation pathways of peroxynitrite in aqueous environments.^44,45^ In contrast, the _(ONOO)_O–O_(water)_ RDF peak at ∼2.8–3.0 Å corresponds to the first solvation shell around ONOO^–^ oxygen atoms, arising from structural packing of water.^46,47^

**Figure 5:**
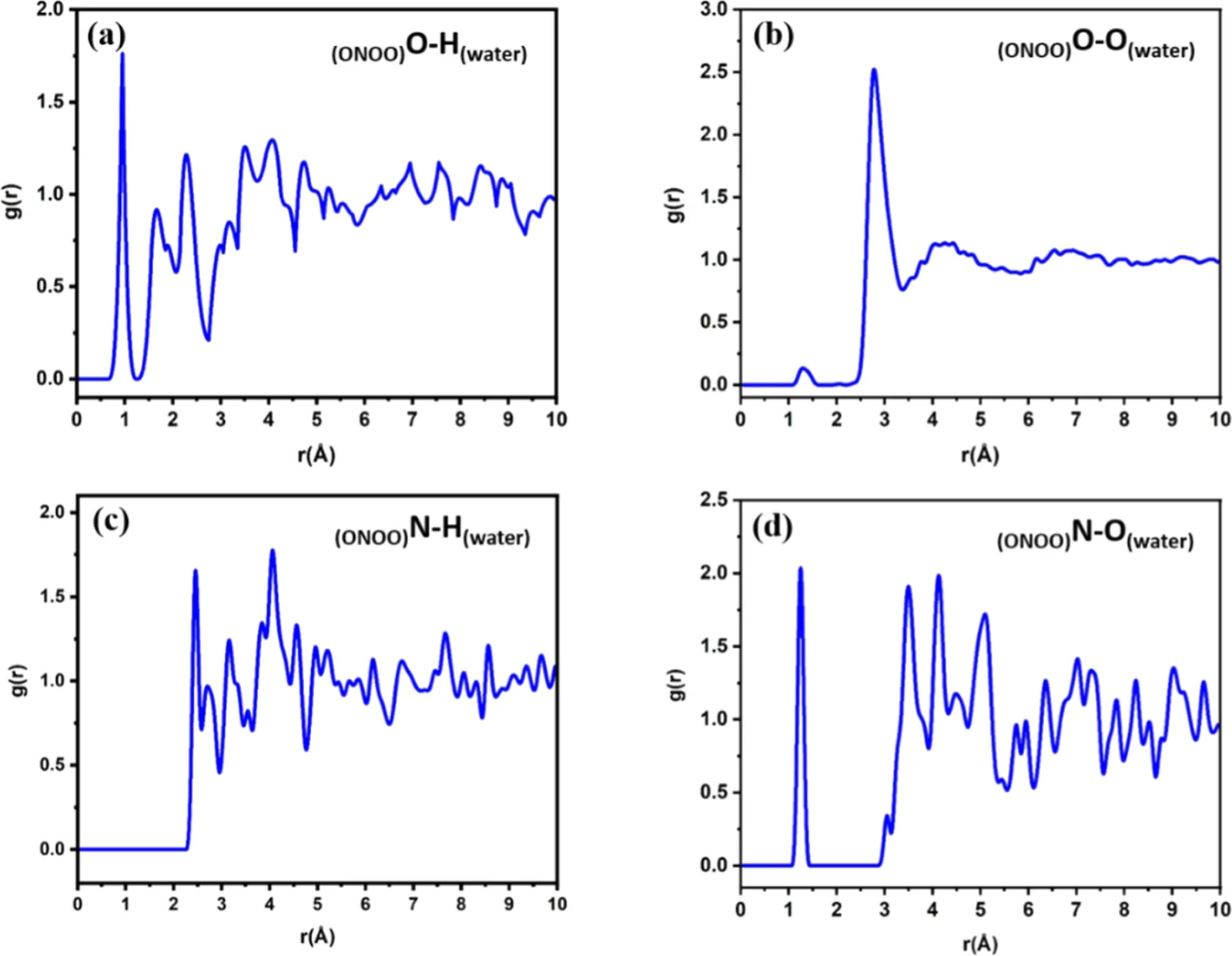
RDFs of ONOO^–^-water interactions at 300 K (5 ONOO^–^ molecules): (a) _(ONOO)_O–H_(water)_, (b) _(ONOO)_O–O_(water)_, (c) _(ONOO)_N–H_(water)_, (d) _(ONOO)_N–O_(water)_.

The _(ONOO)_N–H_(water)_ RDF displays a broader and weaker peak at ∼2.6–2.8 Å, indicating limited interaction strength. This behavior can be attributed to the negatively charged nitrogen site in ONOO^–^, which primarily engages in weak electrostatic or hydrogen-bond-like interactions with surrounding water molecules.^47^ Importantly, the _(ONOO)_N–O_(water)_ RDF shows a distinct peak at ∼1.2–1.3 Å, indicating the formation of new N–O bonds involving water-derived oxygen atoms. This distance is consistent with the typical N=O bond length (∼1.206 Å) reported by Millen and Morton,^48^ suggesting partial chemical transformation of ONOO^–^ toward nitrite– or nitrate– like species in the aqueous phase. The corresponding intermolecular peak at ∼3.2–3.8 Å reflects weaker and more diffuse solvation around the nitrogen atom. Overall, these results demonstrate that both solvation and reactivity are dominated by the oxygen atoms of ONOO^–^, while the nitrogen site exhibits weaker hydration but participates in bond formation through interaction with water-derived oxygen, in agreement with previous theoretical and mechanistic studies.^47^

Complementary to the RDF analysis, the evolution of internal N–O and O–O bond distances within peroxynitrite was tracked to clarify structural changes and reaction pathways. The ONOO^–^ molecule consists of one nitrogen atom and three oxygen atoms (O_1_, O_2_, and O_3_), with an initial connectivity of: O_1_–N–O_2_–O_3_, O_1_, and O_2_ are directly bonded to N, whereas O_3_ is bonded to O_2_, forming the peroxide-like O_2_–O_3_ linkage.

The time evolution of the intramolecular distances in Figure 6a,b shows a clear and selective fragmentation of the ONOO^–^ unit. In Figure 6a, the N–O_1_ distance remains nearly constant at about 1.24–1.28 Å throughout the simulation, while the N–O_2_ distance, after a short initial relaxation from about 1.5–1.6 Å, also stabilizes at 1.24–1.28 Å. The fact that N–O_1_ and N–O_2_ become nearly identical indicates formation of a more symmetric NO_2_^–^ like fragment after dissociation, which is consistent with the expected chemistry of peroxynitrite decomposition and with previous reports describing a nitrogen dioxide-type product after O–O bond cleavage.^26,45,47^ Moreover, the simulated N–O distances are in good agreement with earlier theoretical studies, which reported one short N–O bond in intact ONOO­(H) of about 1.18–1.24 Å and a longer peroxide-side N–O bond prior to cleavage.^26,47^ In contrast, the N–O_3_ distance increases rapidly from its initial bonded value to about 10–35 Å and finally remains near 24–25 Å, demonstrating irreversible separation of O_3_ from the nitrogen-containing fragment. The O–O distances in Figure 6b support the same conclusion. The O_2_–O_3_ distance, corresponding to the initial peroxide bond, increases from about 1.4–1.5 Å to 20–30 Å, clearly showing peroxide-bond cleavage. Likewise, the O_1_–O_3_ distance also becomes large, confirming separation of O_3_ from the residual fragment. By contrast, the O_1_–O_2_ distance remains nearly constant at about 2.0–2.1 Å; although these two oxygen atoms are not directly bonded, this stable separation shows that they remain together within the same nitrogen-containing fragment after loss of O_3_.

**Figure 6:**
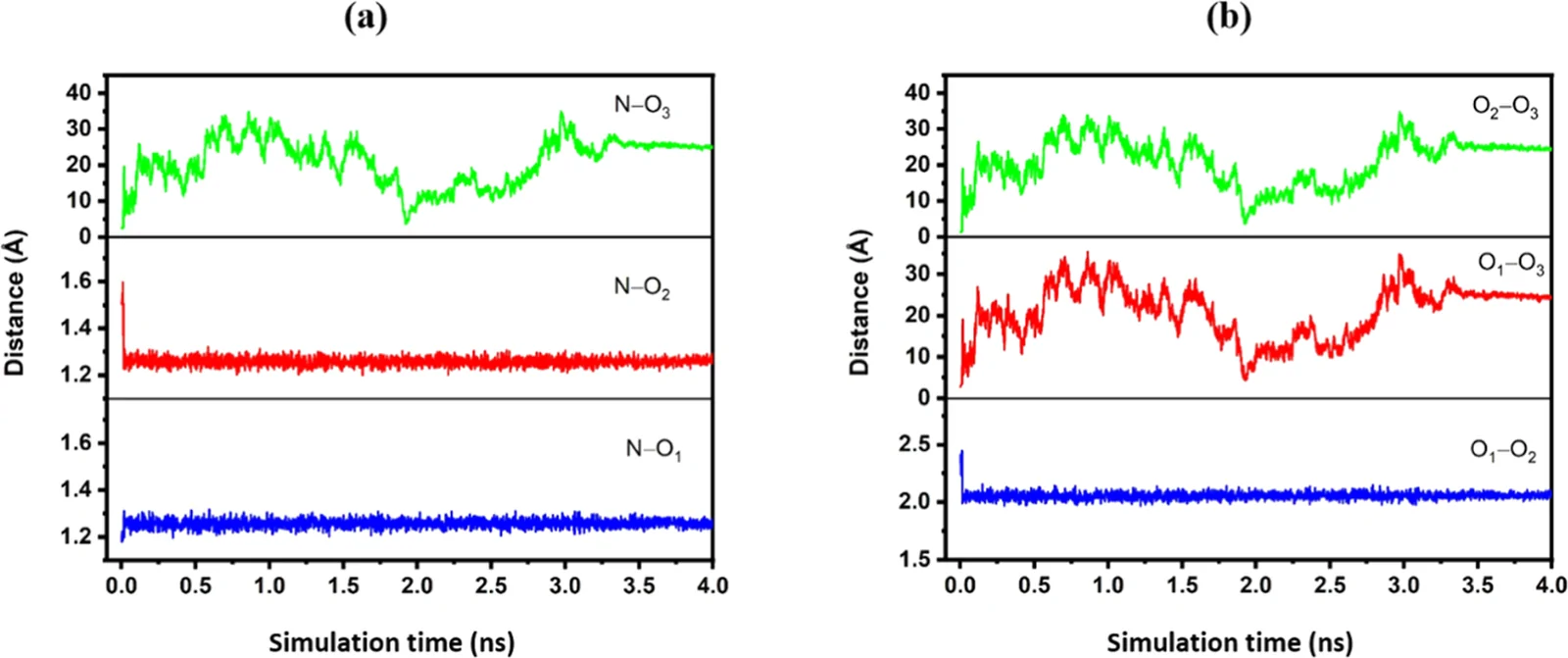
Time evolution of intramolecular (a) N–O and (b) O–O bond distances within peroxynitrite. The results indicate cleavage of the peroxide-side O–O bond, accompanied by separation of O_3_ from the nitrogen-containing fragment.

Taken together, the RDF analysis and the evolution of internal N–O and O–O bond distances demonstrate that water interacts with ONOO^–^ primarily through protonation to form ONOOH, accompanied by preferential solvation at the oxygen sites, particularly around the peroxide moiety. This localized solvation weakens the peroxide linkage and promotes cleavage of the O_2_–O_3_ bond, while the N–O_1_ and N–O_2_ bonds remain intact. These findings are consistent with experimental studies indicating that peroxynitrite undergoes protonation and decomposition via a solvent-caged HO/NO_2_-like intermediate, which may recombine to form nitrate or proceed through alternative pathways, yielding nitrite and oxygen under near-physiological conditions.^45,49^

### Trajectory and Permeation of ONOO^–^ through Liquid Layers

To visualize the motion of peroxynitrite during the MD simulations, only nitrogen-containing molecules were displayed, and water molecules were omitted for clarity. Because ONOO^–^ can react with water and form nitrogen-based intermediates, the analysis was performed by tracking the nitrogen atoms rather than the entire molecules, ensuring a continuous trajectory even when chemical transformations occur. As shown in Figure 7a, the trajectory of a single nitrogen atom is presented as a representative example to illustrate the motion of nitrogen-containing species during the simulation. Only one N atom was selected for visualization because displaying the trajectories of all five nitrogen atoms in the same three-dimensional plot would lead to substantial overlap and reduce the clarity. The plotted trajectory shows the instantaneous positions of the selected nitrogen atom over time, demonstrating that it moves throughout the simulation box after approaching the water phase from its initial position above the liquid layer. This representative behavior indicates active diffusion and continuous redistribution of nitrogen-containing species within the interfacial and aqueous regions during simulation. The other nitrogen atoms exhibit the same overall behavior, although they differ in the regions where they spend more time, with some remaining longer near the middle and others in different parts of the box, while all remain dispersed throughout the simulation volume.

**Figure 7:**
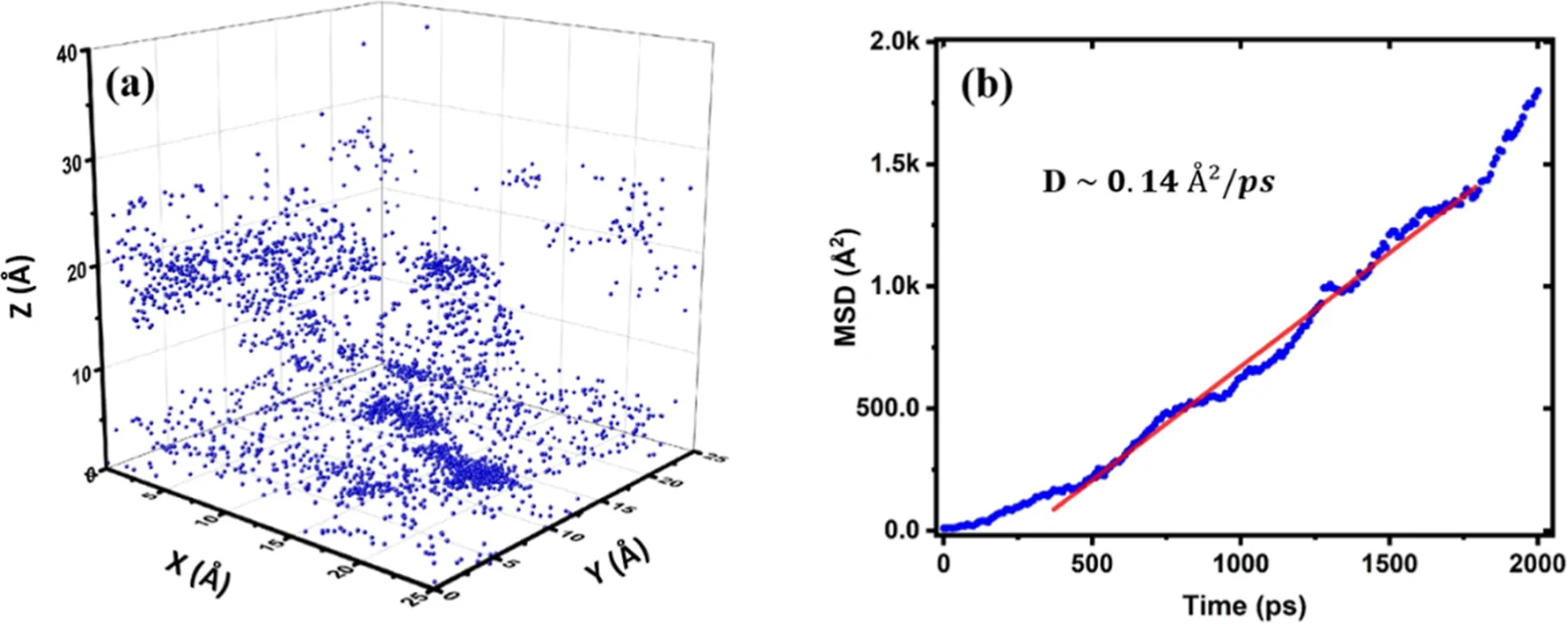
(a) Three-dimensional trajectory of one representative nitrogen atom. Only one N atom is shown for clarity, and water molecules are omitted. (b) MSD of the nitrogen atoms in water at 300 K, with the diffusion coefficient averaged over all five nitrogen atoms using the Einstein relation.

This continuous redistribution reflects the high mobility of these species within the aqueous phase and enables a quantitative evaluation of their transport behavior. The diffusion coefficient was determined directly from the linear region of the MSD curves using the Einstein relation, as described previously, yielding an average value over all five nitrogen atoms of 0.14 Å^2^ ps^–1^, which corresponds to 1.4 × 10^–5^ cm^2^ s^–1^. To the best of our knowledge, there is no study that directly reports the self-diffusion coefficient of peroxynitrite (*D*_ONOO_) from experiment or from an MSD analysis; instead, a recent review estimated *D*_ONOO_ in water at 310.15 K by assuming it to be equal to that of nitrate, giving 2.0 × 10^–5^ cm^2^  s^–1^.^50^ To estimate the value at 300 K, the Stokes–Einstein equation, $D=\frac{k_{B}T}{6\pi \eta R_{hyd}}$ , can be used, which implies *D* ∝ *T*/η if the hydrodynamic radius is assumed constant.^51^

Thus, $D_{300}\approx D_{310}\left(\frac{300}{310.15}\right)\left(\frac{\eta_{310}}{\eta_{300}}\right)$ and using standard water viscosities at 300 and 310 K^52^ gives *D*_300_ ≈ 1.5 × 10^–5^ cm^2^ s^–1^. The diffusion coefficient obtained from the present MD simulation, *D* ≈ 1.4 × 10^–5^ cm^2^ s^–1^, is therefore in good agreement with the available literature estimate, especially considering that the reported literature value is indirect and not based on a direct measurement of ONOO^–^ diffusion.^50^ This consistency supports the validity of our simulation setup and the accuracy of the ReaxFF_CHONx_-2026 force field under ambient conditions.

## Conclusions

In this study, a new reactive force field, ReaxFF_CHON*x*_-2026, was developed and systematically validated to describe the chemistry of RNSs in aqueous environments. The parametrization, based on DFT reference data and representative reaction pathways, addresses key deficiencies of previous models, particularly in capturing nitrogen-centered reactions and bond dissociation processes.

The force field was evaluated through structural, energetic, and dynamic properties of water and reactive species. RDFs confirmed an accurate description of liquid water structure, while the calculated diffusion coefficient (∼0.12 Å^2^ ps^–1^) and thermodynamic consistency demonstrate reliable representation of bulk water behavior. In addition, the potential energy profile of the N–O bond showed close agreement with DFT results, confirming the improved description of bond dissociation compared to earlier ReaxFF parametrizations.

However, extending the application of ReaxFFCHONx-2026 to model more complex biological systems, such as DNA or enzymes, may require additional parametrization to explicitly account for specific interactions with NOx radicals.

The capability of the model to describe nitrogen species in aqueous systems was further demonstrated through molecular dynamics simulations of NO. The results show that NO remains chemically stable in water, with a preserved N–O bond and a diffusion coefficient of ∼0.34 Å^2^ ps^–1^, consistent with literature values. Structural analysis reveals weak, nonreactive solvation dominated by interactions at the oxygen site, confirming that NO forms a stable hydration shell without disrupting the hydrogen-bond network of water.

Reactive simulations of peroxynitrite (ONOO^–^) highlight the importance of water in controlling reactivity. The results indicate that protonation, site-specific solvation, and selective weakening of the peroxide bond govern the decomposition. Bond analysis demonstrates cleavage of the O–O linkage while maintaining the N–O framework, leading to NO_2_-like products, which is in agreement with established mechanisms.

Overall, this work establishes a robust and transferable reactive force field for NO_*x*_- water systems. It provides a foundation for large-scale simulations of plasma-liquid interactions and supports future extensions toward more complex reaction networks relevant to PAW chemistry and related applications.

## Supplementary Material



## Notes

Comparison of ONOOH and direct HNO_3_ formation pathways using ReaxFFCHONx-2026; RDF of NO obtained from MD simulations; definitions and overall MAE/RMSE error metrics; and reaction-energy comparison between ReaxFFCHONx-2026 and DFT. The ReaxFFCHON*x*-2026 parameter set and example LAMMPS simulation files are publicly available at https://github.com/MasoomShaban/ReaxFFCHONx-2026 (PDF)
