# Supplementary material for: A New Reactive Force Field for NO x ‑Water Interactions: Development, Evaluation, and Molecular Dynamics Simulation of Peroxynitrite
Source: J Phys Chem B. 2026 Sep 25;130(40):10540–50. doi: 10.1021/acs.jpcb.6c03657 (PMC13656739; doi:10.1021/acs.jpcb.6c03657)
Supplement: Supplementary file 1 [file jp6c03657_si_001.pdf]

Supporting information for

A New Reactive Force Field for NO_x-Water Interactions: Development, Evaluation, and Molecular Dynamics Simulation of Peroxynitrite

Masoom Shaban[1,2], Adri C. T. van Duin[3], Diana van Duin[3], Margaret Kowalik[3], Mohammad E. Izadi[4], Alfred P. Weber[2], Nina Merkert[1]*

Corresponding Author: masoom.shaban@tu-clausthal.de

[1] Institute of Metallurgy, Clausthal University of Technology, Clausthal-Zellerfeld 38678, Germany

[2]Institute of Particle Technology, Clausthal University of Technology, Clausthal-Zellerfeld 38678, Germany

[3]RxFF Consulting LLC, 1524 West College Ave, Suite 202, State College, Pennsylvania 16801, United States

[4]Department of Chemistry, University of Isfahan, Isfahan 81746-873441, I. R. Iran

Availability of the ReaxFFCHONx-2026 Force Field and Example Files

The complete ReaxFFCHONx-2026 parameter set developed and used in this work is publicly available through GitHub at https://github.com/MasoomShaban/ReaxFFCHONx-2026. The repository also provides an example $ONOO^{-}$–water simulation, including the corresponding LAMMPS input and data files, to facilitate reproducibility and implementation. The force-field parameter file can be used directly with the ReaxFF implementation available in LAMMPS.

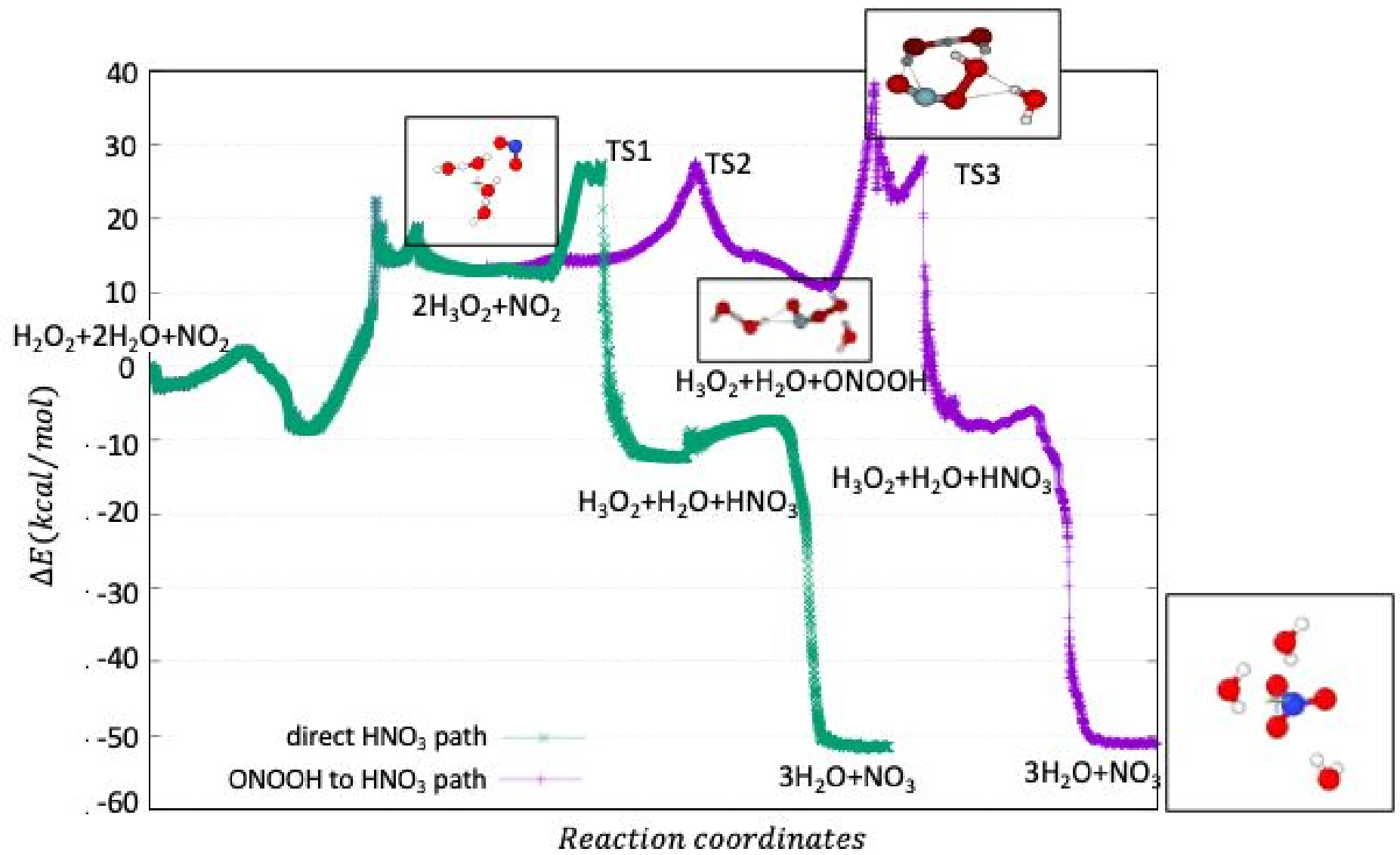

Figure S1 Comparison of ONOOH and direct HNO_3 path using $ReaxFF_{CHONx}$-2026 for conversion to HNO_3 + H_3O. The change in the energy of the system is given as a function of the distance between the water oxygen and nitrogen atom.

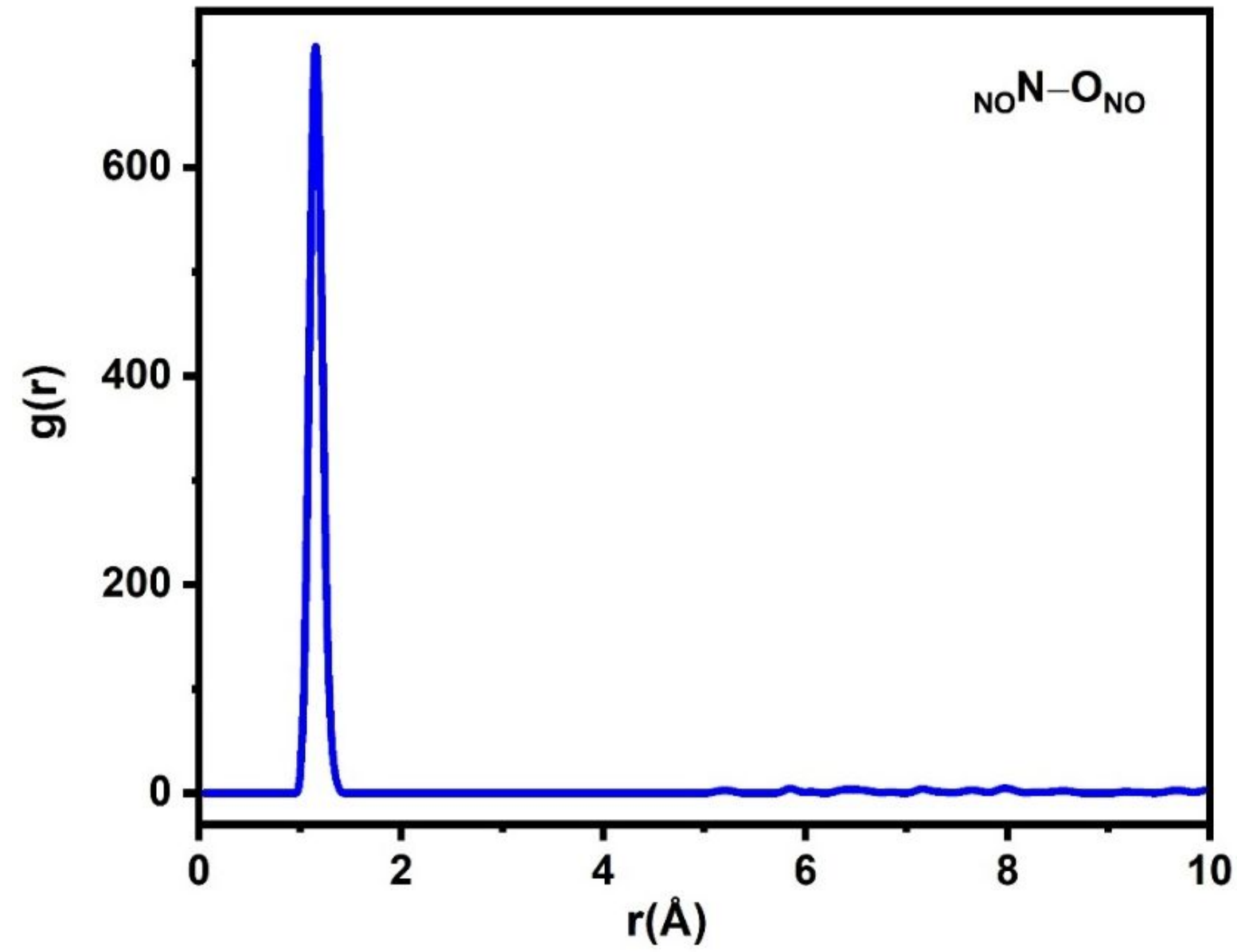

Figure S2 RDF of NO obtained from MD simulations using the $ReaxFF_{CHONx}$-2026

Error definitions: Error = E(ReaxFF) - E(DFT); Absolute error = |Error|; Squared error = $Error^2$.

Overall MAE and RMSE are calculated from all listed reaction energies.

Overall error metrics:

Dataset	n	MAE ($kcal.mol^{-1}$)	RMSE ($kcal.mol^{-1}$)
All Table S1 reaction energies	29	12.59	15.98

Table S1. Reaction-energy comparison between $ReaxFF_{CHONx}$-2026 and DFT. Molecular structures are shown in Figure 1b; L1 and L2 denote model alkene chains with one and two C=C bonds, respectively. All energies are reported in kcal mol^{-1}.

No.	Reaction	ReaxFF E	DFT E	Error = E(ReaxFF) - E(DFT)	|Error|	$Error^2$
**Dissociation / reference bonds**						
1	$N_2 \rightarrow N^{\bullet} + N^{\bullet}$	-234.3344	-224.7	-9.634	9.634	92.82
2	$NO^{\bullet} \rightarrow N^{\bullet} + O^{\bullet}$	-155.4654	-140	-15.465	15.465	239.18
3	$O_2 \rightarrow O^{\bullet} + O^{\bullet}$	-117.4016	-130	12.598	12.598	158.72

No.	Reaction	ReaxFF E	DFT E	Error = E(ReaxFF) - E(DFT)	|Error|	Error²
4	$HOOH \rightarrow OH^{\bullet} + OH^{\bullet}$	-48.2944	-55	6.706	6.706	44.97
11	$\frac{1}{2}N_2O_5 + \frac{1}{2}H_2O \rightarrow HNO_3$	0.9945	-3.93	4.925	4.925	24.25
**Gas-phase NOx / ROS**						
5	$N^{\bullet} + O_2 \rightarrow NO^{\bullet} + O^{\bullet}$	38.0639	15	23.064	23.064	531.94
6	$O^{\bullet} + N_2 \rightarrow NO^{\bullet} + N^{\bullet}$	-78.869	-128.1	49.231	49.231	2423.69
7	$NO^{\bullet} + O^{\bullet} \rightarrow NO_2$	89.7177	103.08	-13.362	13.362	178.55
8	$NO^{\bullet} + \frac{1}{2}O_2 \rightarrow NO_2$	31.0169	38	-6.983	6.983	48.76
9	$O_3 + NO^{\bullet} \rightarrow O_2 + NO_2$	68.9817	56.05	12.932	12.932	167.23
17	$O_3 \rightarrow O_2 + \frac{1}{2}O_2$	37.9648	42.56	-4.595	4.595	21.12
**NOx acid / peroxynitrite-related**						
10	$NO_2 + OH^{\bullet} \rightarrow HNO_3$	42.6437	43.83	-1.186	1.186	1.41
12	$NO^{\bullet} + OH^{\bullet} \rightarrow HNO_2$	47.2119	64.17	-16.958	16.958	287.58
13	$NO_2 + OH^{\bullet} \rightarrow HOONO$	14.6076	29.14	-14.532	14.532	211.19
14	$NO^{\bullet} + HO_2^{\bullet} \rightarrow HOONO$	41.4251	41.52	-0.095	0.095	0.01
15	$NO^{\bullet} + HO_2^{\bullet} \rightarrow HNO_3$	69.4613	71.55	-2.089	2.089	4.36
16	$HOONO + H_2O \rightarrow NO_2 + OH^{\bullet} + H_2O$	-14.6076	-29.17	14.562	14.562	212.06
**Aqueous / ionic NOx**						
18	$NO_2 + H_2O \rightarrow H_2NO_3$	-10.9738	-33.7	22.726	22.726	516.48
19	$NO_2^- + N_2O \rightarrow NO_3^- + N_2$	70.2709	46.07	24.201	24.201	585.68
20	$NO_2^- + O_3 \rightarrow O_2 + NO_3^-$	82.9431	71.28	11.663	11.663	136.03
21	$NO_2 + H_2O \rightarrow \frac{1}{2}NO_3^- + HNO_2 + H_3O^+$	-72.2071	-74.855	2.648	2.648	7.01
**Organic model / lipid-like**						
22	$L1 + OH^{\bullet} \rightarrow L1^{\bullet} + H_2O$	-41.69	-37.53	-4.160	4.160	17.31
23	$L1^{\bullet} + O_2 \rightarrow L1\_OO^{\bullet}$	-18.1322	-21.42	3.288	3.288	10.81
24	$L1^{\bullet} + L1\_OO \rightarrow L1\_OOH + L1^{\bullet}$	-13.5968	1.56	-15.157	15.157	229.73
25	L1_OO → L1_2OH	20.8135	24.61	-3.796	3.796	14.41
26	$L2\_OO + NO^{\bullet} \rightarrow L2\_ONOO$	-41.4024	-29.01	-12.392	12.392	153.57
27	L2_O → L2_e	4.3661	-10.88	15.246	15.246	232.44
28	$L2\_O + NO^{\bullet} \rightarrow L2\_ONO$	-30.1408	-48.33	18.189	18.189	330.85
29	$L2 + HO_2^{\bullet} \rightarrow L2\_OOH$	-25.7635	-48.56	22.797	22.797	519.68
